# Depression, distress and self-efficacy: The impact on diabetes self-care practices

**DOI:** 10.1371/journal.pone.0175096

**Published:** 2017-03-31

**Authors:** Cassidy Devarajooh, Karuthan Chinna

**Affiliations:** Department of Social and Preventive Medicine, Faculty of Medicine, University of Malaya, Jalan Universiti, Wilayah Persekutuan, Kuala Lumpur, Malaysia; Institute of Endocrinology and Metabolism, ISLAMIC REPUBLIC OF IRAN

## Abstract

The prevalence of type 2 diabetes is increasing in Malaysia, and people with diabetes have been reported to suffer from depression and diabetes distress which influences their self-efficacy in performing diabetes self-care practices. This interviewer administered, cross sectional study, conducted in the district of Hulu Selangor, Malaysia, involving 371 randomly selected patients with type 2 diabetes, recruited from 6 health clinics, aimed to examine a conceptual model regarding the association between depression, diabetes distress and self-efficacy with diabetes self-care practices using the partial least square approach of structural equation modeling. In this study, diabetes self-care practices were similar regardless of sex, age group, ethnicity, education level, diabetes complications or type of diabetes medication. This study found that self-efficacy had a direct effect on diabetes self-care practice (path coefficient = 0.438, p<0.001). Self-care was not directly affected by depression and diabetes distress, but indirectly by depression (path coefficient = -0.115, p<0.01) and diabetes distress (path coefficient = -0.122, p<0.001) via self-efficacy. In conclusion, to improve self-care practices, effort must be focused on enhancing self-efficacy levels, while not forgetting to deal with depression and diabetes distress, especially among those with poorer levels of self-efficacy.

## Introduction

Diabetes mellitus is a common chronic disease in Malaysia. According to national studies, the prevalence of diabetes has increased from 11.6% in 2006 to 15.2% in 2011 [1]. People with diabetes suffer from a higher burden of psychosocial problems and psychological disorders [2]. The prevalence of depression is higher among people with diabetes, and is partly attributed by vascular damage which may induce cerebral pathology that constitutes vulnerability for depression [3]. Depression adds to the burden of managing diabetes, as those with depression perform poorer diabetes self-care [4].

Diabetes distress, an affective disorder, is a syndrome comprised of a multidimensional component, such as worry, conflict, frustration, and discouragement that can accompany living with diabetes, and is closely related to depression [5]. Majority of people with diabetes who were depressed experienced diabetes distress, however, most of those experiencing diabetes distress were not depressed [6]. Diabetes distress effects an individual’s problem solving skill which is required to carry out diabetes self-care and this may result in poorer self-care practices, and ultimately poorer glycemic control [7].

Both depression [8]and diabetes distress [9] influences self-efficacy. A high level of self-efficacy is needed to manage the daily challenges associated with caring for diabetes. Individuals with higher levels of self-efficacy perform better diabetes self-care practices [10, 11].

**Fig 1:**
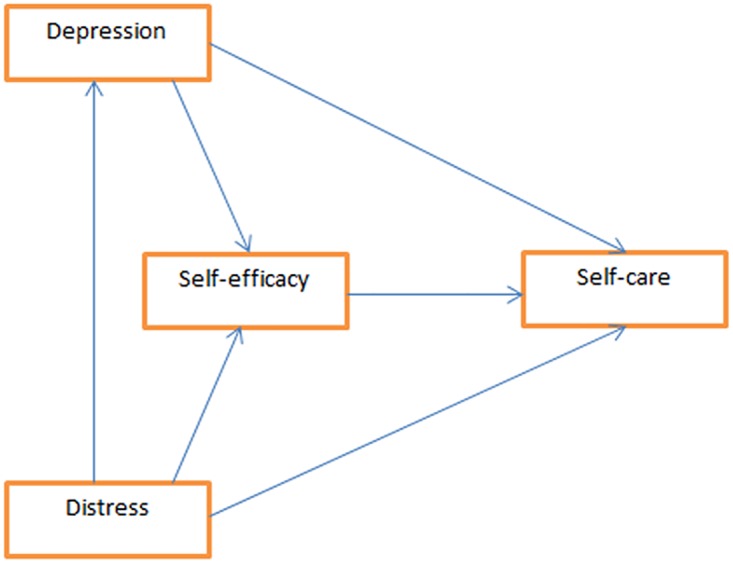
Hypothesized model of the relationship between depression, diabetes distress and self –efficacy with self-care practices.

This study aims to explore the relationship between depression, diabetes distress and self-efficacy with diabetes self-care practices. Up to date, there has been no study in Malaysia which assessed the structural relationship between depression, diabetes distress and self-efficacy with diabetes self-care practices. Based on the available literature, it is hypothesized that self-efficacy affects diabetes self-care directly, while depression and diabetes distress both have direct and indirect effects via self-efficacy on diabetes self-care. Diabetes distress is hypothesized to affect depression directly. Fig 1 illustrates the relationship between self-care with depression, self-efficacy and distress.

## Materials and methods

This was a cross sectional study with patients recruited from all 6 primary health clinics in the district of Hulu Selangor. Hulu Selangor is the biggest district in the state of Selangor, measuring about 174,047 hectares. In the district of Hulu Selangor, there were 6,396 patients with type 2 diabetes receiving treatment in 6 government health clinics. The fees for each visit inclusive of consultation, investigation and medication is only 1 Ringgit Malaysia (1 US Dollar = 4.40 Ringgit Malaysia, average exchange rate in December 2016). Eligibility criteria as a participant in this study was being a Malaysian older than 18 years old, having a diagnosis of type 2 diabetes and can at least understand the Malay language. The exclusion criteria were pregnancy and cognitive impairment such as dementia or mental retardation. Eligible patients were approached for written consent for the study.

### Sample size

To perform a partial least square structural equation modeling, Henseler et al. [12] recommended a 10 to 1 ratio of sample size to model parameter. The theoretical model in this study had 6 model parameter. Thus with a ratio of 10 to 1, the sample size required was 60. For a reliable analysis, a minimal sample size of 200 is considered good. To determine diabetes self-care practice, the sample size was calculated using the Open Epi software version 3.01. With a type 2 diabetic population of 6,396, power of 80%, confidence interval of 95% and an anticipated frequency of good self-care at 52% [13], the required sample size was 361. The sample size was increased by 30% after considering non-responders. The final sample size was 480 patients. A proportionate number of patients were selected from all 6 clinics based on the number of patient attendees. Every 10th patient on the follow-up list was approached to participate in this study. The purpose and relevance of the study was explained to all the potential participants.

### Data management and statistical analysis

This was an interviewer-based study. Data was double entered and cleaned before analysis. The Statistical Package for the Social Science (SPSS) version 20 was used for descriptive and inferential analysis and subsequently for factor analysis of the diabetes distress scale.

Categorical variables were presented as frequency (n) and percentages (%), and numerical variables were presented as mean and standard deviation (SD). ANOVA and t test were used to determine differences in continuous data.

For factor analysis, a good factor analytical model must have several properties. The guidelines provided by Hair et al (2010) were used [14]. The item correlation must be between 0.3–0.9, the Kaiser-Meyer-Olkin (KMO)>0.6 and the factor loading ideally >0.7 but not less than 0.5 is acceptable. The preferred Average variance extracted (AVE) is >50%, but may be less if some factor loading are <0.7. The correlation between subdomains of the diabetes distress scale should be<0.85 to have discriminant validity.

The Smart PLS3 software was used for the structural equation modeling. The path coefficients were calculated between variables and the significance level was set as p<0.05.

### Ethical issue

Ethical approval for this study was obtained from Malaysian Institute of Public Health, registration number NMRR-13-93-15292 and from the University of Malaya Ethical Committee. Permission to conduct the study was also obtained from the State Health Director, District Heath Offices and the respective Medical Officers.

#### Self-care

Diabetes self-care was assessed using the Malay version of the Summary of Diabetes Self Care Activities scale. The Malay version of the Summary of Diabetes Self Care Activities scale has 12 items and measures levels of diabetes self-care in 5 major areas; diet, exercise, adherence to medication, blood glucose testing and foot care. Every item measures the number of days each diabetes self-care activities were practiced in the last seven days, and is scored between 0 to 7. To assess overall diabetes self-care, the score of all items was divided with the total number of items. Similarly, to assess self-are for individual areas of diabetes care, the score of all items within the respective area of diabetes care was divided with the corresponding number of items. From a possible score of between 0 to 7, a score of 4 and above was considered as good practice [15, 16].

#### Self-efficacy

Self-efficacy was assessed using the Malay version of the Diabetes Management Self Efficacy scale [15]. This questionnaire has 14 items and measures self-efficacy in 4 major areas; diet, exercise, medication adherence and blood glucose control. Each item was scored between 0 to 10. The scoring of this scale was based on the total score of all items, with higher score indicating better self-efficacy. There was no scoring for individual areas of self-efficacy. The possible score for this scale was between 0 to 140.

#### Depression

Depression was assessed using the Malay version of the PHQ (Patient Health Questionnaire)– 9 item questionnaire. The Patient Health Questionnaire-9 (PHQ-9) is a self-report measure to screen for depression, consisting of nine questions with each item being scored from 0 to 3. The PHQ-9 is scored by calculating the total score of all 9 items. The total PHQ-9 scores ranges from 0 to 27 with the scores of 10 or more categorized as depression [17].

#### Diabetes distress

Diabetes distress was assessed using the Diabetes Distress Scale (DDS), a 17 item questionnaire, measuring 4 domains of distress; [18] emotional burden, physician related distress, regimen related distress and interpersonal distress. Each item was measured on a Likert Scale of 1–6, where higher values indicate distress. The DDS allows overall distress or the individual domain to be measured. The score for the scale was based on the average score of all the items, with a possible score of between 1 to 6. A score of 3 or more was categorized as distress. This questionnaire was not available in the Malay language. Originally in the English language, it was translated and validated in this study

### Data collection tools and measurements

The consented eligible patients were required to complete the survey. A face-to-face interview was conducted. Self reported data regarding socio-demographics, diabetes self-care practices, self-efficacy, depression and diabetes distress were collected by trained interviewers.

### Translation, pre-test and pilot test

Originally in English and not available in the Malay language, the Diabetes Distress Scale was translated and validated prior being used. The scale has 17 items, measuring distress in four domains; emotional burden, physician related distress, regiment related distress and interpersonal distress. As the scale was not available in the Malay language, it underwent a translation process. The forward translation was performed by 3 individuals while the backwards translation by 2 individuals. All translators were familiar and fluent with the original language and target language. A panel decided on the best translation that suited the target population. The translated version underwent pre-testing involving 16 patients with diabetes and pilot testing involving 40 patients with diabetes recruited from the same study setting. All the domains had good internal consistency with Cronbach Alpha >0.7.

## Results

Four hundred and eighty eligible patients were approached, 391 agreed to participate in this study, giving a response rate of 81.5%. The sex, age, duration of diabetes and HbAc1 values were comparable between responder and non-responder (Refer Table 1)

**Table 1:** Comparison of baseline characteristics between responder and non-responder.

Variables	Mean ± SD		p—value
	Responder (391)	Non–Responder (89)	
Sex*	Male(145) Female(246)	Male(40) Female(49)	0.169
**Age (years)**	55.33 ± 10.09	55.71 ± 12.63	0.762
**Duration of diabetes (years)**	6.02 ± 4.60	5.26 ±4.04	0.153
**HbA1c (%)**	8.77 ±2.29	8.50 ± 1.96	0.308

t-test was performed for continuous data

* Chi-square performed with a significance level set at <0.05.

After data cleaning, only 371 participants were included for analysis. The mean age of the participants was 55.33 ± 10.09 years. Among the 371 study participants, 141 (38.0%) were males, 215(58.0%) were of Malay ethnicity followed by Indians at 110 (29.6%) and Chinese at 46 (12.4%).

Majority of the study participants, n = 189 (50.9%) attained primary education (≤6 years of formal education), followed by secondary education (7–11 years of formal education), n = 149 (40.2%) and lastly tertiary education (≥12 years of formal education) n = 33 (8.9%). The most prescribed oral hypoglycemic agent was biguanide, n = 340 (91.6%), followed by sulphonyurea, n = 251 (67.7%), acarbose n = 18 (4.9%) and lastly glitazones, n = 9 (2.4%). One hundred and three (27.8%) study participants were prescribed insulin injections. Among the study participants, 41 (11.4%) had retinopathy, 21 (5.8%) had ischemic heart disease, 4 (1.1%) had stroke while 2 (0.6%) had nephropathy.

The mean diabetes self-care score was 3.87 ± 0.82, with 170 (45.8%%) categorized as practicing overall good diabetes self-care. For the individual self-care domains, medication adherence had the highest score, followed by foot care, diet, exercise and lastly self-monitoring of blood glucose, with the respective scores being 6.01 ± 1.98, 5.63 ± 1.84, 4.70 ± 1.56, 2.77 ± 1.78 and 1.38 ± 1.59. Among the 371 study participants, 303 (81.7%) practiced good medication adherence, 290 (78.2%) practiced good foot care, 266 (71.7%) practiced good diet, 112 (30.2%) had good exercise practices and lastly 32 (8.6%) had good self-monitoring of blood glucose practices.

The mean depression score was 4.58 ± 2.57, with 16(4.3%) categorized as depressed. The mean diabetes distress score was 1.54 ± 0.66, with 20 (5.4%) categorized as distressed. The mean self-efficacy score was 104.08 ± 23.20, from a possible score of between 0 to 140. (Refer Table 2)

**Table 2:** Participants demographics, self-care and psychosocial factors.

Characteristics	Mean ± SD	N (%)
**Age (years)**	55.33 ± 10.09	
**Sex**		
Male		141 (38.0%)
Female		230 (62.0%)
**Race**		
Malay		215(58.0%)
Chinese		46 (12.4%)
Indian		110 (29.6%)
**Education level**		
Primary education (1–6 yrs)		189 (50.9%)
Secondary education (7–11 yrs)		149 (40.2%)
Tertiary education (≥12 yrs)		33 (8.9%)
**Medication type**		
Biguanide		340 (91.6%)
Sulphonyurea		251 (67.7%)
Acarbose		18 (4.9%)
Glitazone		9 (2.4%)
Insulin		103 (27.8%)
**Complications of diabetes**		
Ischemic Heart Disease		21 (5.8%)
Stroke		4 (1.1%)
Nephropathy		2 (0.6%)
Retinopathy		41 (11.4%)
**SDSCA score (self-care)**	3.87 ± 0.82	
Good practice		170 (45.8%)
Poor practice		201 (54.2%)
**Diet score**	4.70 ± 1.56	
Good practice		266 (71.7%)
Poor practice		105 (28.3%)
**Exercise score**	2.77 ± 1.78	
Good practice		112 (30.2%)
Poor practice		259 (69.8%)
**Medication adherence score**	6.01 ± 1.84	
Good practice		303 (81.7%)
Poor practice		68 (18.3%)
**SMBG score**	1.38 ± 1.59	
Good practice		32 (8.6%)
Poor practice		339 (91.4%)
**Foot care score**	5.63 ± 1.98	
Good practice		290 (78.2%)
Poor practice		81 (21.8%)
**PHQ score (depression)**	4.58 ± 2.57	
Depressed		16(4.3%)
Non-Depressed		355 (95.7%)
**DDS score (distress)**	1.54 ± 0.66	
Distressed		20 (5.4%)
Non-Distressed		350 (94.6%)
**DMSE score (self-efficacy)**	104.08 ± 23.20	

The self-care practices were similar regardless of sex, age, ethnicity, education level, diabetes complications and diabetes medication. Similarly, depression was not influenced by sex, age, ethnicity, education level, diabetes complication or diabetes medication. Self-efficacy level was significantly higher among those with secondary education level when compared to those with primary education level. Self-efficacy was similar between sex, age group, ethnicity, complication status and diabetes medication. Diabetes distress was significantly higher among the Malays when compared to the Indians and among those with tertiary education when compared to those with primary education. Diabetes distress level was similar between sex, age group, complication status and diabetes medication. (Refer Table 3).

**Table 3:** Socio-demographic characteristics and its associated factors among the study participants.

Characteristics, n(%)	Self-care		Self-efficacy		Diabetes distress		Depression	
	Mean ± SD	P value	Mean ± SD	P value	Mean ± SD	P value	Mean ± SD	P value
**Sex**								0.645
Male, 141(38.0%)	3.79 ± 0.77	0.181	104.51 ± 21.34	0.777	1.62 ± 0.71	0.066	4.50 ± 2.54	
Female, 230(62.0%)	3.91 ± 0.70		103.81 ± 24.30		1.49 ± 0.62		4.63 ± 2.60	
**Ethnicity**								0.681
Malay, 215(58.0%)	3.82 ± 0.90	0.392	103.86 ± 25.29	0.535	1.63 ± 0.72	0.007^b^	4.67 ± 2.85	
Chinese, 46(12.4%)	3.92 ± 0.67		101.21 ± 21.69		1.46 ± 0.67		4.35 ± 2.16	
Indian, 110(29.6%)	3.94 ± 0.68		105.70 ± 19.22		1.39 ± 0.47		4.50 ± 2.12	
**Age**								0.776
≤60 years, 255(68.7%)	3.92 ± 0.70	0.071	105.25 ± 22.75	0.15	1.53 ± 0.66	0.970	4.61 ± 2.62	
>60 years, 116(31.3%)	3.77 ± 0.70		101.51 ± 24.05		1.54 ± 0.66		4.46 ± 2.44	
**Education level**						0.008^c^		0.245
Primary, 189(50.9%)	3.78 ± 0.74	0.080	101.46 ± 23.53	0.012^a^	1.49 ± 0.62		4.74 ± 2.70	
Secondary, 149(40.2%)	3.98 ± 0.85		108.05 ± 21.09		1.53 ± 0.65		4.32 ± 2.29	
Tertiary, 33(8.9%)	3.90 ± 1.00		101.18 ± 28.06		1.88 ± 0.84		4.91 ± 2.98	
**Complication status**		0.913		0.425		0.286		1.00
With at least one complication, 67(18.1%)	3.86 ± 0.78		102.03 ± 23.31		1.63 ± 0.77		4.58 ± 2.79	
No complication, 304(81.9%)	3.87 ± 0.82		104.53 ± 23.18		1.52 ± 0.64		4.58 ± 2.52	
**Diabetes treatment**		0.056		0.929		0.071		0.491
OHA only, 268(72.2%)	3.81 ± 0.81		104.32 ± 23.27		1.52 ± 0.62		4.60 ± 2.47	
Insulin only, 18(4.9%)	4.21 ± 0.85		104.50 ± 20.95		1.89 ± 0.94		3.89 ± 2.76	
OHA and Insulin, 85(22.9%)	3.97 ± 0.82		103.23 ± 23.64		1.54 ± 0.71		4.67 ± 2.83	

^a^ secondary education—primary education = 6.59

^b^ Malays—Indians = 0.231

^c^ tertiary education—primary education = 0.384

**Fig 2:**
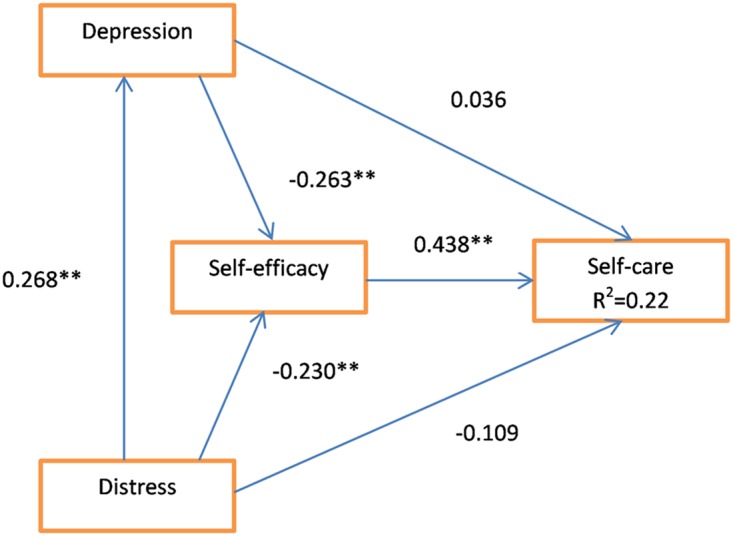
Relationship between depression, diabetes distress and self –efficacy with self-care practices. **p<0.001.

To assess the relationship between self-care with self-efficacy, depression and diabetes distress as illustrated in Fig 1, a partial least square structural equation modeling analysis was performed using the SmartPLS 3 software. As indicated in Fig 2, there was a significant direct positive effect from self-efficacy (path coefficient = 0.438, p<0.001) to diabetes self-care. There were also significant direct negative effects from depression (path coefficient = -0.263, p<0.001) and from diabetes distress (path coefficient = -0.230, p<0.001) to diabetes self-efficacy. There was a significant positive effect from diabetes distress (path coefficient = 0.268, p<0.001) to depression. Both depression and diabetes distress had no significant direct association with self-care, but had significant negative indirect effect on self-care, via self-efficacy. The indirect effect of depression (path coefficient = -0.115, p<0.001) and distress (path coefficient = -0.122, p<0.001) indicate that depressed and distressed individuals had lower self-efficacy and performed poorer self-care. This model explained 22% of variation in self-care. (Refer Table 4)

**Table 4:** Direct and indirect effects.

Outcome	Direct effect	Indirect effect	Total effect
**Self –care**			
Self-efficacy →self-care	0.438**		0.438
Depression → self-care	0.036	-0.115**	-0.079
Distress → self-care	-0.109	-0.122**	-0.231
**Self-efficacy**			
Depression → self-efficacy	-0.263**		-0.263**
Distress → self-efficacy	-0.230**	-0.070*	-0.300**
**Depression**			
Distress → depression	0.268**		0.268*

*p<0.01,

**p<0.001

The response to the 17 items of diabetes distress scale was subjected to principal axis factoring to test the dimensionality of the items in the construct [19]. Items which had poor convergence (correlation <0.3) with the construct or poor discriminance (correlation >0.9) were removed. Items with poor factor loading (<0.5) were also removed. In the final version of the questionnaire, the emotional burden domain had 4 items (item number 4 removed due to poor correlation), the physician related distress domain had 3 items (item number 9 removed due to poor factor loading), the regimen related distress domain had 4 items (item number 14 removed due to poor factor loading) and the interpersonal domain had 3 items. The sample size was adequate with as the KMO values were >0.6, and had good convergence validity as the AVE were around or more than 50% (Refer Table 5). All pairwise correlations between the constructs were less than 0.85. Hence, there is sufficient discriminant validity between the construct (Refer Table 6).

**Table 5:** Factor analysis of the diabetes distress scale.

Items	Factor loading			
	Emotional burden	Physician related distress	Regimen related distress	Inter -personal distress
1. Feeling that diabetes is taking up too much of my mental and physical energy every day.				
2. Feeling angry, scared, and/or depressed when I think about living with diabetes.	0.769			
3. Feeling that diabetes controls my life.	0.634			
^a^ 4. Feeling that I will end up with serious long-term complications, no matter what I do.	0.524			
5. Feeling overwhelmed by the demands of living with diabetes.	0.749			
6. Feeling that my doctor doesn't know enough about diabetes and diabetes care.		0.796		
7. Feeling that my doctor doesn't give me clear enough directions on how to manage my diabetes.		0.722		
8. Feeling that my doctor doesn't take my concerns seriously enough.		0.723		
^b^ 9. Feeling that I don't have a doctor who I can see regularly enough about my diabetes.		0.488		
10. Feeling that I am not testing my blood sugars frequently enough.			0.728	
11. Feeling that I am often failing with my diabetes routine.			0.802	
12. Not feeling confident in my day-to-day ability to manage diabetes.			0.654	
^c^ 13. Feeling that I am not sticking closely enough to a good meal plan.			0.469	
14. Not feeling motivated to keep up my diabetes self-management.			0.771	
15. Feeling that friends or family are not supportive enough of self-care efforts (e.g. planning activities that conflict with my schedule, encouraging me to eat the "wrong" foods).				0.657
16. Feeling that friends or family don't appreciate how difficult living with diabetes can be.				0.794
17. Feeling that friends or family don't give me the emotional support that I would like.				0.816
**Correlation matrix**	0.325–0.587	0.527–0.571	0.405–0.708	0.520–0.648
**KMO (Kaiser-Meyer-Olkin) value**	0.741	0.705	0.742	0.696
**AVE (Average variance extracted)**	48%	56%	55%	58%
**Initial items**	5	4	5	3
**Final items**	4	3	4	3

^a^ removed (correlation = 0.272)

^b,c^ removed (Factor loading <0.5)

**Table 6:** Correlation between domains of the translated diabetes distress scale.

	Correlation between constructs			
Construct	Emotional	Physician	Regimen	Interpersonal
Emotional	-			
Physician	0.619	-		
Regimen	0.793	0.692	-	
Interpersonal	0.677	0.617	0.816	-

## Discussion

As has been shown from the pilot study and the factor analysis, the translated Diabetes distress Scale was valid. Factor analysis was performed to ensure that the translated Diabetes distress Scale measures what it was meant to while enabling the items to be reduced into a smaller set to save time and facilitate easier interpretation [20]. The final translated Diabetes Distress scale has 14 items. All the items within the respective domains had item correlation of between 0.3–0.9, which meant that all the items measured the same underlying theory. The KMO (Kaiser-Meyer-Olkin) value for all domains of the translated Diabetes Distress scale were > 0.6, indicating that the sample size was sufficient to perform a factor analysis. Only one factor was extracted from each domain, with the factor loading of all items being >0.5, indicating good relationship between each item with the underlying factor[20]. All pairwise correlations between the constructs were less than 0.85, indicating that there was no sign of multicollinearity [21].

The baseline characteristics between responder and non-responder were the same, thus reducing any possible responder bias.

Though there is limited information regarding diabetes self-care practices among Malaysians, the finding of this study was in agreement with the available studies which reported non satisfactory diabetes self-care practices [13, 22]. Medication adherence was the most practiced self-care and this was most likely because medication was provided for free by the healthcare provider and unlike diet and exercise which requires lifestyle changes, it was easier to performed [23]. Furthermore, the immediate effect or derangement of health outcome if medication prescription was not followed may increase their compliance rate [24]. Unlike medications which were provided for free, glucose testing machines and their test strips were not provided by the health clinics and patients were required to purchase it themselves. Having to personally finance the cost for self-monitoring of blood glucose may limit the practice [22, 25].

In this study, sex did not influence self-care. Reports regarding the association between sex with self-care has not been consistent [26, 27], and is influenced by the local and traditional sociocultural gender role [28]. The self-efficacy levels were similar between sex in this study. Adebayo et al. [29] and Venkataraman et al. [30] reported that when controlled for social gender role and sociodemographic factors, self-efficacy does not differ between sex. This study found no association between sex with diabetes distress or depression. Previous studies have reported that females were more likely to experience diabetes distress and depression [4, 31, 32] and this has been attributed to their biological nature, difference in mood and gender roles [33, 34].

This study found no association between age with self-care, and similar findings have been reported by other studies [35–38]. Age was not associated with self-efficacy, depression and diabetes distress in this study. This finding was in agreement with previous studies which reported that age was not associated with psychosocial factors, but instead other factors such as sociodemographic level, social support, comorbidity and overall well-being were [39–42].

The self-care practices, self-efficacy and depression were similar between ethnicities. Diabetes distress was lowest among the ethnic minority. This finding did not conform to previous finding reported elsewhere. Though there is no specific information regarding Malaysian diabetics, study elsewhere have associated ethnic minorities as marginalized groups with issues concerning access to healthcare services [43]. Ethnic minorities have also been reported to face more socioeconomic constraints, poor education and perceived discrimination, thereby increasing the levels of depression and diabetes distress [4, 44]. In this study, all the participants regardless of ethnicity or socioeconomic status had equal access to healthcare services.

The education level did not influence diabetes self-care practices. Previous studies reported that those with higher education performed better self-care as they had better awareness [45]. In this study, all, the participants had equal access to healthcare services. Self-efficacy was higher among those with higher education. This finding was in agreement with previous studies [46, 47]. This study found higher levels of diabetes distress among those with tertiary education. The association between education level with diabetes distress has not been consistent as other factors such as employment and income which are closely related to education influences distress as well [48, 49].

In this study, diabetes complications were not associated with diabetes self-care, self-efficacy, distress or depression. Previous studies have reported that patients with diabetes experiencing more or severe complications were associated with poorer self-care, lower self-efficacy, depression and diabetes distress [31, 50]. In this study, the severity of the complications were not explored, which may have influenced the current finding.

This study found that the type of medication prescribed did not influence diabetes self-care, self-efficacy, diabetes distress or depression. Other studies have reported that those on insulin exercised lesser due to the fear of hypoglycemia [51] and were more likely to be non-compliant to insulin injections as it causes discomfort and interferes with their daily activities [52]. The complexity of insulin therapy has been reported to result in poorer self-efficacy [53] especially concerning the proper timing and dosages, while the discomfort and interference of daily activities associated with insulin injections has been regarded as burdening by some, resulting in significant emotional distress [54] and a higher prevalence of depression among insulin users [54, 55].

Using partial least square to assess structural relationship, this study found that self-efficacy was a strong predictor of diabetes self-care. The positive association between self-efficacy and diabetes self-care practices was in agreement with previous studies [56–58]. High levels of self-efficacy is associated with better self-autonomy, more confidence, more initiative and persistence in dealing with daily needs of diabetes care [59]. A higher level of self-efficacy also ensures the continuity of appropriate diabetes self-care practices [60]. A high sense of self-efficacy amplifies and strengthens an individual’s well-being in many ways. Individuals with confidence in their capabilities looks at difficult tasks as challenges to be overcome rather than a problem to be avoided [61].

Contrary to what we expected, in this study, depression did not influence self-care practices. This finding could have been due to the low prevalence of depression and difference in study population [62]. Furthermore, the almost free health services among the participants of this study could have served as a protective factor from depression [63]. Similarly, in this study, diabetes distress did not influence self-care practices. This finding could have been due to the low prevalence of distress among the study participants and the sociocultural background of the participants. Previous studies have reported that socio-cultural norms influences ones perception of disease. Studies have shown that Asians and Caucasians perceive disease differently [64]. Though not having any direct association with self-care, both depression and diabetes distress were indirectly associated with self-care via self-efficacy. Diabetics experiencing depression and distress had lower levels of self-efficacy and later practiced poorer self-care. Diabetics experiencing distress have been reported to have lower levels of self-efficacy [65], while depression leads to problems such as apathy, hopelessness, fatigue, memory problems and loss of confidence in performing daily activities which are all required in managing a chronic disease like diabetes [66, 67].

The positive association between distress with depression was in agreement with previous studies [68, 69]. Diabetes distress is caused by the difficulty in coping with diabetes in daily life. A minimal amount of diabetes distress is part of living with diabetes. However, when severe enough, or exacerbated by other environmental or personal factor, diabetes distress maybe severe enough to lead to depression [68].

Overall, among the study population, self-efficacy was the most important factor in determining good diabetes self-care. Though depression and diabetes distress affected self-care indirectly via self-efficacy, the prevalence of these conditions were low. Thus, to improve self-care practices, effort must be focused on enhancing self-efficacy levels, while not forgetting to deal with depression and diabetes distress, especially among those with poor self-efficacy.

## Conclusion

This study is the first in our knowledge to explore the relationship between depression, diabetes distress and self-efficacy with self-care practices among Malaysians with type 2 diabetes. Having higher levels of self-efficacy was associated with better diabetes self-care practices. Furthermore, managing depression and diabetes distress is important among diabetics as it may lead to poor self-efficacy and subsequently poorer diabetes self-care. Based on the insights gained from this study, future research should focus on the same topic, with more emphasis on increasing patient’s self-efficacy level and to reduce depression and diabetes distress with the ultimate aim of improving diabetes self-care practices.

## Limitations

There are limitations to this study that should be acknowledged. Firstly, the result of this study represents the population under study, which are people with type 2 diabetes who were being cared for in government healthcare centers in the district of Hulu Selangor. Therefore, the results should not be generalized and needs to be replicated in different patient groups. Secondly, the questionnaires utilized in this study were self-reported. Thirdly, this study was of a cross sectional study design, thus no statement of causality can be made.

## Supporting information

S1 File Raw data file. (XLSX)
